# How to achieve Tat transport with alien TatA

**DOI:** 10.1038/s41598-017-08818-w

**Published:** 2017-08-18

**Authors:** René Steffen Hauer, Roland Freudl, Julia Dittmar, Mario Jakob, Ralf Bernd Klösgen

**Affiliations:** 1 Institute of Biology – Plant Physiology, Martin Luther University Halle-Wittenberg, Halle/Saale, 06099 Germany; 2 Institut für Bio- und Geowissenschaften 1, Biotechnologie, Forschungszentrum Jülich GmbH, 52425 Jülich, Germany

## Abstract

TatA is an essential and structurally conserved component of all known Twin-arginine transport (Tat) machineries which are able to catalyse membrane transport of fully folded proteins. Here we have investigated if bacterial TatA, or chimeric pea/*E. coli* TatA derivatives, are capable of replacing thylakoidal TatA in function. While authentic *E. coli* TatA does not show any transport activity in thylakoid transport experiments, TatA chimeras comprising the transmembrane helix (TMH) of pea TatA are fully active. For minimal catalytic activity it is even sufficient to replace three residues within TMH of *E. coli* TatA by the corresponding pea residues. Almost any further substitution within TMH gradually raises transport activity in the thylakoid system, while functional characterization of the same set of TatA derivatives in *E. coli* yields essentially inverse catalytic activities. Closer inspection of the substituted residues suggests that the two transport systems have deviating demands with regard to the hydrophobicity of the transmembrane helix.

## Introduction

The twin-arginine translocation (Tat) pathway, which is found at the thylakoid membrane of chloroplasts and the plasma membranes of bacteria and archaea (for recent reviews see refs 1–4), is specifically engaged by proteins carrying signal peptides with a characteristic twin pair of arginine residues within their N-region which gave rise to the name of the pathway^5, 6^. The energy for their membrane transport is provided solely by the transmembrane potential, notably ΔpH and/or ΔΨ^7, 8^.

The Tat pathway is unique in its ability to translocate fully folded proteins across ion-tight membranes^9–12^. It permits the co-transport of prosthetic groups or cofactors, like iron-sulphur clusters or molybdopterin, together with their apoproteins across the lipid bilayer^13–15^, which might have been the fundamental cause for the development and evolutionary persistence of this transport pathway.

The Tat machinery of chloroplasts and Gram-negative bacteria consists of three subunits, namely TatA, TatB, and TatC (in the thylakoid system also called Tha4, Hcf106, and cpTatC, respectively)^16^. TatC is a polytopic protein with six transmembrane helices and an N-terminal stromal/cytosolic domain^17^. Together with TatB, which carries a single N-terminal membrane anchor^18^, it constitutes the oligomeric TatBC receptor which binds precursor proteins carrying twin-arginine signal peptides^19–21^. The actual membrane translocation of the passenger protein additionally requires the transmembrane potential and the presence of TatA^22^, a membrane protein with strikingly similar structure and membrane topology as TatB^18, 23^. However, while TatB is generally found together with TatC in the heteromeric membrane receptor complexes of approximately 560–700 kDa^19, 24, 25^, the role of TatA in the transport process is still enigmatic. In a prevalent model TatA is assumed to constitute membrane pores of different or variable diameter facilitating the translocation of passenger proteins of different size^22, 26, 27^. Alternatively, it was proposed that the recruitment of TatA to the substrate-loaded Tat receptor would lead to a thinning or weakening of the lipid bilayer in the vicinity of the folded transport substrate which in turn would permit translocation of the passenger directly across the lipid phase^28^. And finally, a catalytic or regulatory activity of TatA exhibiting cooperative effects in the translocation process was demonstrated^29^ which might be indicative for a function of TatA as co-enzyme that transforms the TatBC receptor complex into the active translocase.

In line with its yet unresolved mode of operation, the stoichiometry of TatA remains a matter of debate. In *E. coli* an excess of TatA over TatB and TatC is generally assumed^30^, while in the plant system the stoichiometry of the Tat subunits is still contested. Both substoichiometric^25^, stoichiometric^25, 31^, as well as excess amounts of TatA^32^ compared with TatB and TatC were described depending on the method used for analysis and/or the plant species studied.

Remarkably, even the localisation of TatA is ambiguous to some extent. Though being described as membrane protein in all systems analysed, it was also found in soluble form in the stroma of chloroplasts^33^ as well as in the cytosol of *B. subtilis* ^34^. For chloroplasts it was shown that stromal TatA can fully substitute the thylakoid-bound protein moiety in function^33^. Furthermore, it was possible after suppressing the intrinsic thylakoidal TatA activity by antibody treatment to reconstitute thylakoidal Tat transport by adding soluble TatA obtained from *in vitro* translation or bacterial overexpression^29, 35^. This unique property allowed for the identification of functionally important residues within the polypeptide chain^35^ and the exact quantification of TatA demand during membrane transport of a model Tat substrate^29^.

Here, we have applied this approach to investigate if bacterial TatA, or chimeric pea-*E. coli* TatA derivatives, are likewise capable of replacing thylakoidal TatA in function. While authentic *E. coli* TatA does not show any transport activity in our thylakoid transport experiments, increasing numbers of pea residues within its transmembrane helix (TMH) gradually raises the catalytic activity of the protein suggesting that the entire TMH plays a role in the translocation process. Remarkably, functional characterisation of the same set of TatA derivatives in *E. coli* yields essentially inverse transport characteristics.

## Results

### *E. coli* TatA cannot replace plant TatA in thylakoid transport experiments

One suitable approach to study the activity of TatA in the membrane transport of proteins are *in thylakoido* complementation assays. In such assays the intrinsic activity of TatA is suppressed by antibody treatment and reconstituted by supplementing the assays with TatA obtained from *in vitro* translation or bacterial overexpression^29, 35^. With this approach it was, for example, possible to demonstrate that the intrinsic TatA activity of pea thylakoids can be fully substituted not only by the native protein from pea but also by TatA proteins from heterologous plant species like *Arabidopsis thaliana* ^29^.

**Figure 1:**
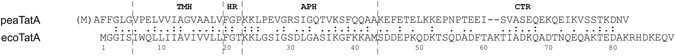
Alignment of TatA proteins from pea and *E. coli*. The amino acid sequences (given in the one-letter-code) of the mature TatA proteins from pea (*peaTatA*) and *E. coli* (*ecoTatA*) were aligned with LALIGN^54^. Identical and conserved residues are indicated by *colons* and *dots*, respectively. The designation of the amino acid positions given below the lanes refers to *E. coli* TatA. The transmembrane helix (*TMH*), hinge region (*HR*), amphipathic helix (*APH*), and unstructured hydrophilic C-terminal region (*CTR*), as deduced from NMR spectroscopy of the T22P derivative of *E. coli* TatA^23^, are indicated. The initial methionine shown in *brackets* for peaTatA is not part of the mature protein in chloroplasts but was added to the clone used for *in vitro* translation.

**Figure 2:**
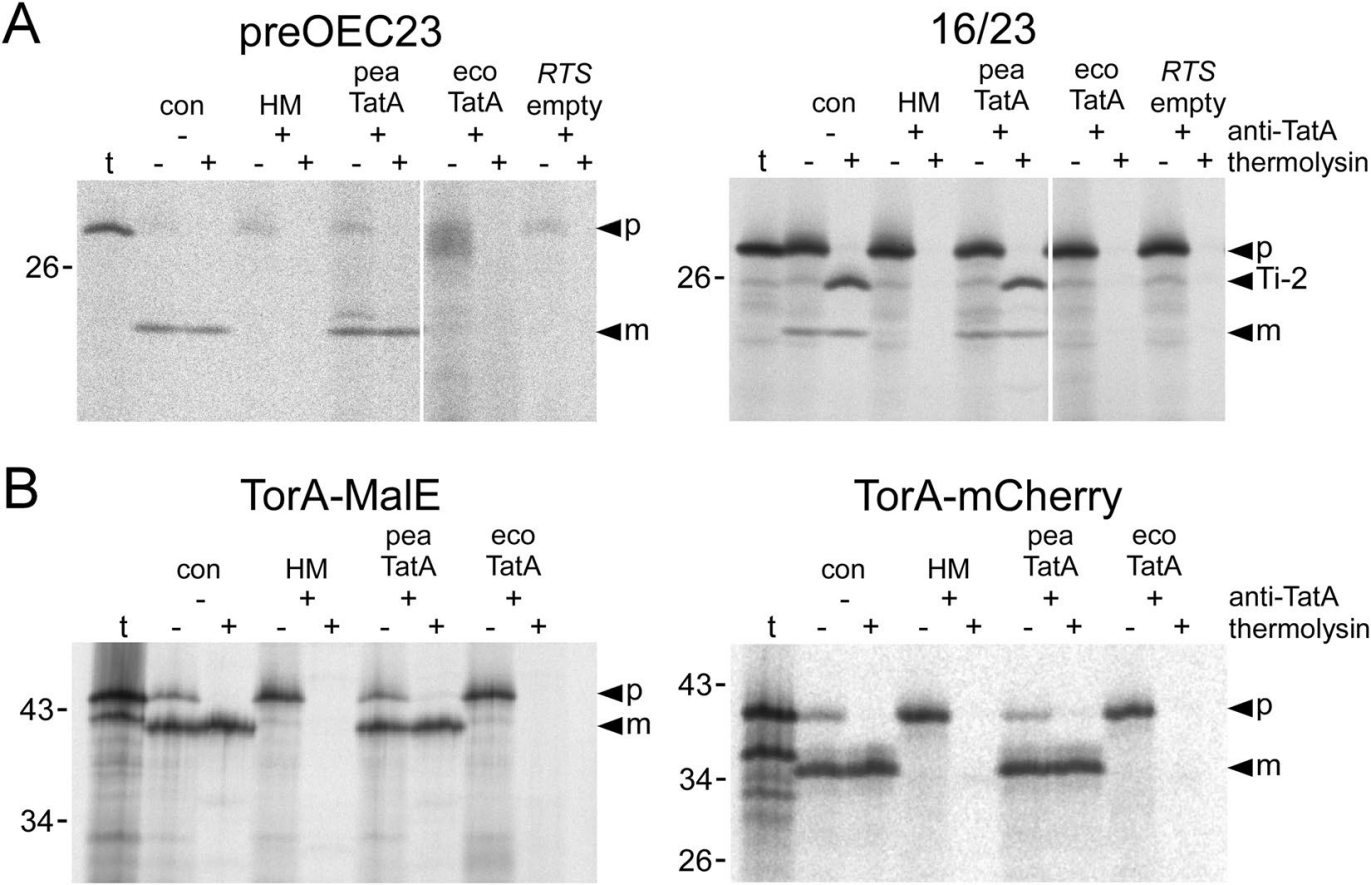
Reconstitution of thylakoidal Tat transport with TatA from pea and *E. coli*. (**A**) *In thylakoido* transport of authentic and chimeric plant model Tat substrates in the presence of TatA from pea or *E. coli*. The authentic precursor of OEC23 (*preOEC*23) and the chimeric precursor protein *16/23* were generated by *in vitro* transcription of the respective cDNA clones and subsequent *in vitro* translation in the presence of [^35^S]-methionine. 5 μl of each translation assay were incubated with thylakoid vesicles isolated from pea that had been either mock-treated (*con*) or treated with antibodies against TatA (+*anti-TatA*). The latter were subsequently supplemented with either HM buffer only (10 mM Hepes/KOH, pH 8.0; 5 mM MgCl_2_) (*HM*) or with TatA from pea (*pea TatA*) or *E. coli* (*eco TatA*), which were obtained by *in vitro* transcription/translation with the wheat germ rapid translation system (*RTS*). Lanes *RTS empty* show translation assays containing an empty vector control. After incubation for 15 min in the light at 25 °C, thylakoids were washed once with HM buffer and subsequently incubated with either thermolysin (182 μg/ml, 30 min on ice, *lanes*+) or HM buffer (*lanes−*). From each fraction stoichiometric amounts corresponding to 7.5 μg of chlorophyll were analysed on 10–17.5% SDS-polyacrylamide gradient gels and detected by phosphorimaging. In *lanes t*, 1 μl of the translation assays of the respective Tat substrates was loaded. The bands showing the precursor (*p*) and mature proteins (*m*) are indicated by *filled arrowheads*. *Ti-2* marks the protease-protected fragment indicative of translocation intermediate *Ti-2* of the 16/23 chimera in which the passenger protein is fully translocated across the membrane but the Tat transport signal not yet removed^24, 48^. The molecular weights (in kDa) of marker proteins loaded in parallel are indicated on the left of each panel. (**B**) *In thylakoido* transport of the bacterial model Tat substrates TorA-MalE and TorA-mCherry.

In contrast, despite remarkable sequence conservation between plant and bacterial TatA proteins, particularly in the membrane interacting regions (Fig. 1), TatA from *E. coli* cannot substitute for the intrinsic thylakoidal TatA activity in protein transport (Fig. 2). This lack of transport activity is not a consequence of substrate selectivity of TatA because neither plant Tat substrates, like the precursor of the 23 kDa subunit of the oxygen evolving system (preOEC23) or the chimeric model substrate 16/23, nor even bacterial Tat substrates, like the chimeric model substrates TorA-MalE or TorA-mCherry^36, 37^, show any membrane transport in these assays (Fig. 2A and B). In contrast, all these proteins are efficiently transported if the assays are complemented with pea TatA which reconfirms earlier observations that also bacterial Tat substrates are principally suited for thylakoidal membrane transport^38, 39^.

**Figure 3:**
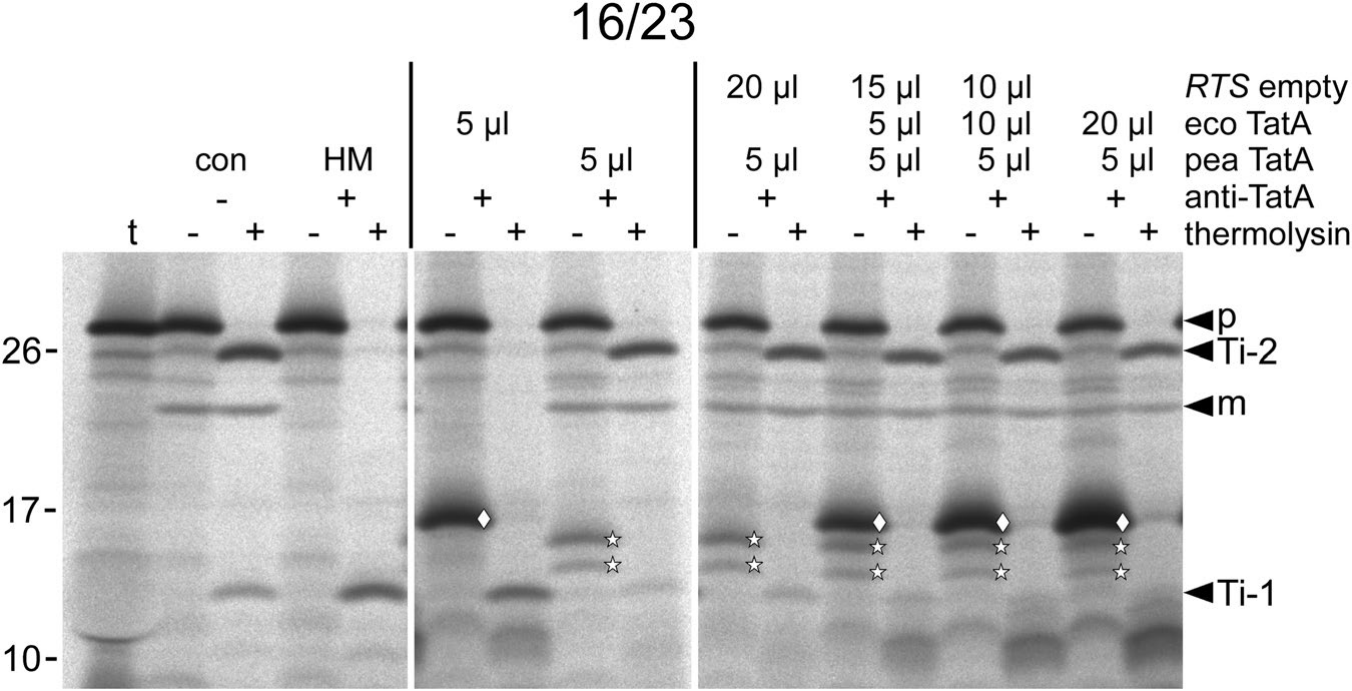
Influence of increasing amounts of ecoTatA on thylakoid transport mediated by peaTatA. *In thylakoido* complementation of 16/23 transport by peaTatA was analysed in the presence of increasing amounts of ecoTatA. Both TatA proteins were obtained from *in vitro* translation in the RTS system in the presence of [^35^S]-methionine, which allows their detection by autoradiography (*stars*: peaTatA, *diamond*: ecoTatA). The composition of each supplementation, i.e. the proportion of pea TatA, eco TatA, and empty RTS in the total volume of 25 μl, is given above the lanes. Ti-1 indicates the position of the protease-protected fragment indicative of translocation intermediate *Ti-1* of the 16/23 chimera which represents the membrane-bound precursor protein prior to membrane translocation of the 23 kDa passenger polypeptide^24, 48, 55^. For further details see the legend to Fig. 2.

One conceivable explanation for the lack of transport activity of *E. coli* TatA in the thylakoid system could be inability of bacterial TatA to bind to the thylakoid membrane, as a consequence, for example, of the unique lipid composition of this membrane^40^. However, we did not find any indication for such incompatibility because *E. coli* TatA shows strong membrane binding when applied in radiolabelled form in our *in thylakoido* experiments (Fig. 3, *E. coli* TatA is indicated by diamonds).

In addition, the presence of *E. coli* TatA in the assays does not impair the general integrity of the thylakoid system, e.g. by destruction of the transthylakoidal proton gradient, which would likewise prevent Tat-dependent protein transport (Fig. S1). If *in thylakoido* assays performed with pea TatA are additionally supplemented with increasing amounts of *E. coli* TatA, membrane transport of Tat substrates like the 16/23 chimera remains essentially unaffected (Fig. 3) which rules out any unspecific damage of the thylakoid membrane by the bacterial protein. Furthermore, it shows that the presence of bacterial TatA in the assays does not have a negative impact on the activity of pea TatA which makes an immediate interaction of the two protein moieties and the formation of functionally relevant heterooligomeric TatA complexes like membrane pores, which presumably would be less active or even inactive, rather unlikely. Instead, it appears that *E. coli* TatA is incapable of productively interacting with the other components of the thylakoidal Tat machinery. Hence, bacterial and plant TatA are apparently too divergent from each other to permit mutual substitution, in spite of a largely conserved structure, as deduced from molecular modelling of TatA from *Arabidopsis thaliana* (Fig. S2) and the NMR structures available for the T22P derivative of *E. coli* TatA^23^.

### The N-terminal transmembrane helix of pea TatA provides full transport activity to chimeric TatA proteins

**Figure 4:**
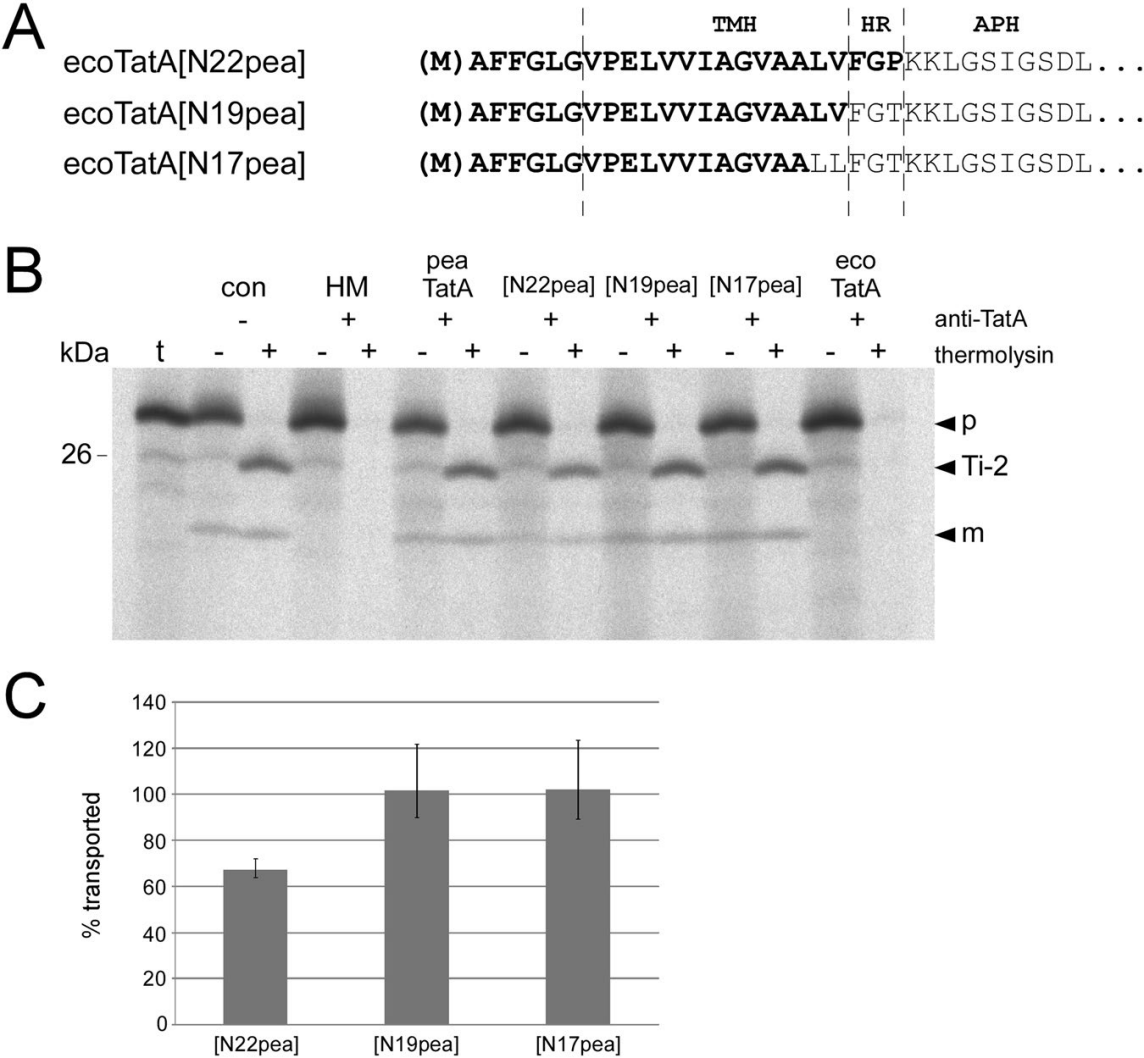
Catalytic activity of chimeric TatA proteins in in thylakoido complementation experiments. (**A**) Amino acid sequence of the N-terminal regions of the chimeric TatA proteins ecoTatA[N22pea], ecoTatA[N19pea], and ecoTatA[N17pea]. The residues derived from pea are depicted in bold. Please note that due to the use of *E. coli* TatA as reference the number of pea residues in each chimera is actually two residues higher than depicted in the name (see also Fig. 1). (**B**) *I* *n thylakoido* complementation of 16/23 transport by the RTS-generated chimeric TatA proteins shown in (*A*). For further details see the legends to Figs 1 and 2. (**C**) Quantitation of catalytic activities as shown in (*B*) given as percentage of the transport rate determined for the antibody-treated assays supplemented with pea TatA. Both mean values and standard deviations from three (n = 3) independently repeated experiments are shown.

As the first step to identify those differences that are functionally relevant, we have generated a set of chimeric TatA proteins in which segments from pea and *E. coli* TatA were combined. In chimera ecoTatA[N22pea], the N-terminal 22 residues of *E. coli* TatA comprising the transmembrane helix (TMH) and the short hinge region (HR) connecting TMH and the amphipathic helix (APH) were replaced by the corresponding residues of pea TatA (Fig. 4A). In chimera ecoTatA[N19pea], solely TMH but not HR originate from the pea protein. And in chimera ecoTatA[N17pea], even the C-terminal residue of TMH was retained from *E. coli*.

All three chimeric TatA proteins show strong transport activity in the *in thylakoido* complementation assays (Fig. 4B) demonstrating that the large C-terminal unstructured region, and even APH, of pea TatA can be replaced by the corresponding regions of the *E. coli* protein without loss of function. However, while the activity of both ecoTatA[N17pea] and ecoTatA[N19pea] is essentially identical to that of pea TatA, ecoTatA[N22pea] is always found somewhat less active (approximately 65% of the transport activity of pea TatA, Fig. 4C). This was entirely unexpected because from all three chimeras ecoTatA[N22pea] carries the largest proportion of pea TatA. It should be noted though that the HRs of *E. coli* and pea TatA, and hence the chimeras ecoTatA[N19pea] and ecoTatA[N22pea], differ in only a single amino acid residue at position 22, which is T and P, respectively (Fig. 4A). Remarkably, such a T22P substitution, which was also introduced into the *E. coli* TatA derivative used for NMR analysis^23^, was already described to render *E. coli* TatA functionally inactive^41^. Together with our results it appears that such a T22P substitution always has a negative impact on TatA activity if combined with the APH and/or the C-terminal unstructured region of *E. coli* TatA, irrespective of whether it is analysed in bacterial or thylakoidal transport systems.

### Successive substitution of pea TMH residues leads to stepwise reduction of TatA activity

**Figure 5:**
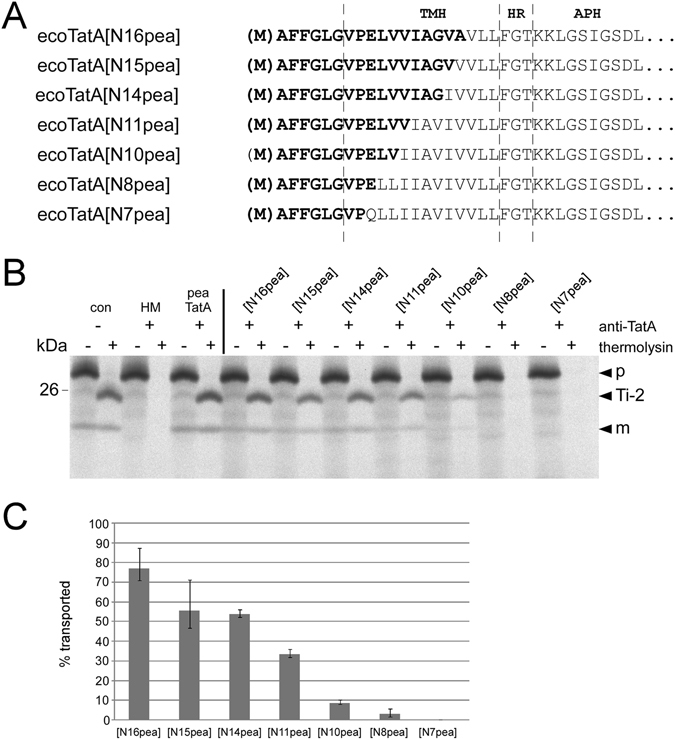
Catalytic activity of chimeric TatA proteins with substitution in the C-terminal part of the TMH region. (**A**) Amino acid sequence of the N-terminal regions of the chimeric TatA proteins analysed here. (**B**) *In thylakoido* complementation assays analysing the chimeric TatA proteins shown in (*A*). (**C)** Quantitation of catalytic activities of three (n = 3) independent experiments as shown in (*B*) given as percentage of the transport rate determined for the antibody-treated assays supplemented with pea TatA. For further details see the legends to Figs 1, 2 and 4.

In the next set of TatA chimeras, the proportion of pea residues was further reduced. Starting from the fully active chimera ecoTatA[N17pea], the pea-derived residues within TMH were successively substituted from C- to N-terminus by the respective residues of the *E. coli* protein (Fig. 5A). This leads to a gradual decrease of catalytic TatA activity in *in thylakoido* complementation experiments (Fig. 5B). While ecoTatA[N16pea] still shows 77% of the activity of pea TatA, virtually each additional substitution reduces the activity further until it is, for chimera ecoTatA[N7pea], completely abolished (Fig. 5C). This gradual decrease of transport activity suggests that there are not only a few key residues but rather residues along the entire TMH of TatA that determine the species-specific functionality of a TatA molecule in a given Tat system.

### Minimal TatA activity demands for three residues from pea TatA

**Figure 6:**
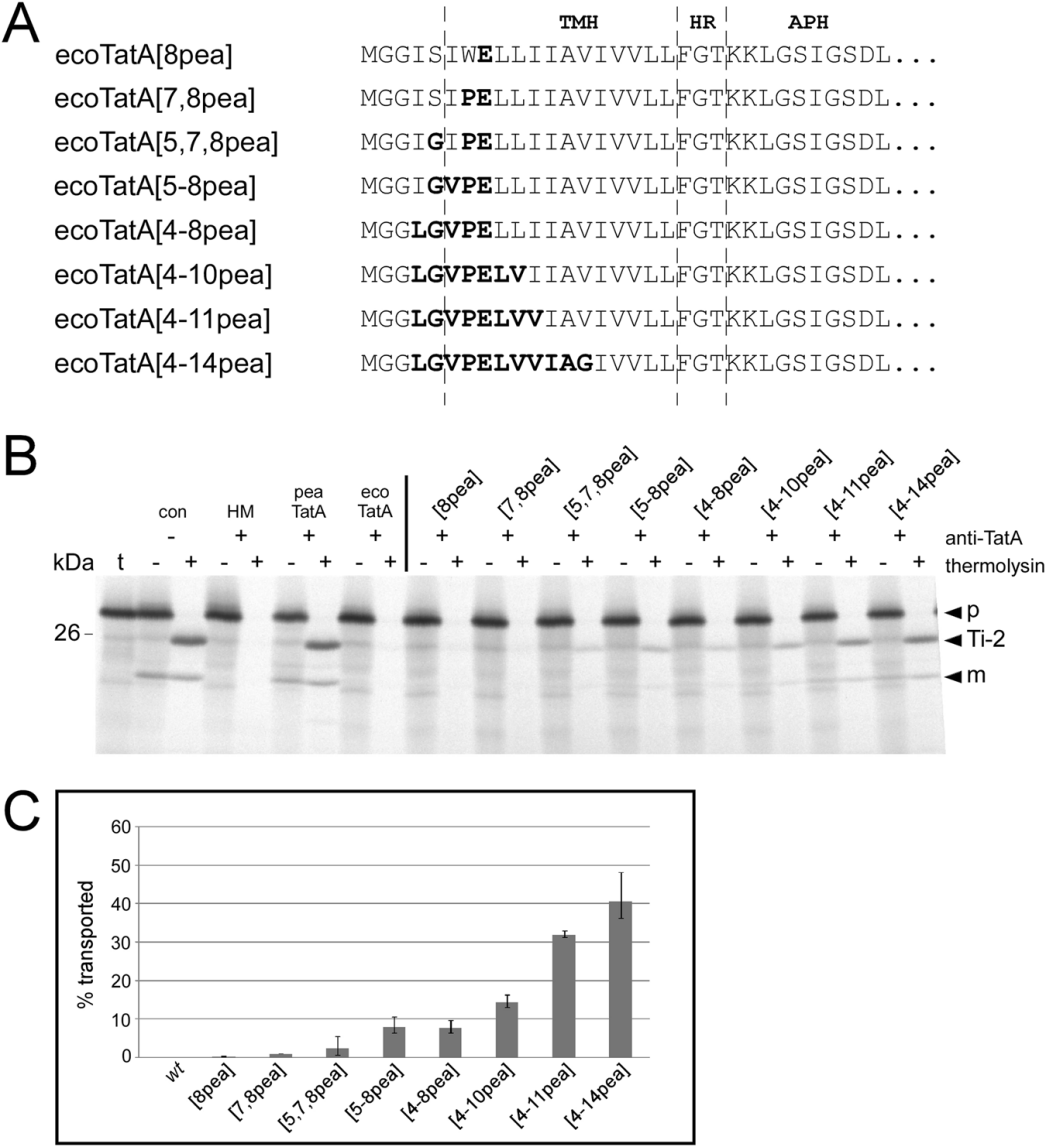
Catalytic activity of mutant derivatives of *E. coli* TatA carrying single amino acid substitutions within TMH. (**A**) Amino acid sequence of the N-terminal regions of the chimeric TatA proteins analysed here. (**B**) I*n thylakoido* complementation assays analysing the chimeric TatA proteins shown in (*A*). (**C**) Quantitation of catalytic activities of three (n = 3) independent experiments as shown in (*B*) given as percentage of the transport rate determined for the antibody-treated assays supplemented with pea TatA. For further details see the legends to Figs 1, 2, and 4.

In order to determine the impact of N-terminally located residues independent of changes in the C-terminal part of the TMH, a further set of TatA mutants was generated in which single amino acids within *E. coli* TatA were substituted by the corresponding residues from pea (Fig. 6A). Analysing these mutants in *in thylakoido* complementation assays, it turned out that neither the replacement of the glutamine residue at pos. 8 by glutamic acid (chimera ecoTatA[8pea]), which is an essential residue in plant TatA proteins^29, 35^, nor the additional substitution of tryptophan at pos. 7 by proline in chimera ecoTatA[7,8pea] leads to any detectable TatA activity (Fig. 6B). Only after further replacement of serine at pos. 5 by glycine (chimera ecoTatA[5,7,8pea]), thylakoid transport of the 16/23 model substrate can be observed, albeit at a minimal level (approx. 3% of the control, Fig. 6C). Additional replacement of isoleucine by valine at pos. 6 (ecoTatA[5–8pea]) leads to a substantial increase of transport activity (to approx. 8%), whereas substitution of isoleucine at pos. 4 by leucine (ecoTatA[4–8pea]) has no additional effect. However, each of the next two substitutions (leucine to valine at pos. 10 and isoleucine to valine at pos. 11 in mutants ecoTatA[4–10pea] and ecoTatA[4–11pea], respectively) almost doubles the transport activity (to approximately 15% and 30%, respectively) (Fig. 6C). And finally, mutant ecoTatA[4–14pea], which additionally comprises a substitution of valine to glycine at pos. 14, shows more than 40% of the catalytic activity of the authentic TatA protein from pea.

Hence, a minimal substitution of three residues in the N-terminal region of TMH of *E. coli* TatA (S5G, W7P, Q8E) is both required and sufficient to establish low but definite catalytic TatA activity in the *in thylakoido* assays. Successive replacement of further residues within the TMH by the corresponding residues from pea TatA leads to stepwise increase of the transport activity in the thylakoid system which finally can reach 40–50% of the transport activity of authentic pea TatA (Figs 5C and 6C).

### Activity of the TatA chimeras in *E. coli*

Next, we have asked which of the chimeric TatA proteins generated here are capable of catalysing Tat transport in *E. coli*. For this purpose, the authentic *tat*A gene in plasmid pHSG-TatABC, which is expressed under control of the *lac* promoter, was replaced by the respective chimeric TatA encoding genes. The resulting plasmids were transformed into *E. coli* GSJ101 (*ΔmalE*, *ΔtatABCE*) that, in addition, contained plasmid pTorA-MalE from which the strictly Tat-specific TorA-MalE reporter protein is expressed (Table 1). As described earlier, the TorA-MalE reporter allows an easy *in situ* detection of Tat-dependent MalE export into the periplasm on indicative media, i.e. growth on minimal maltose medium (MMM) and formation of red colonies on MacConkey maltose (MCM) agar plates^42, 43^.

**Table 1:** Phenotype of bacterial strains on maltose minimal medium and MacConkey maltose.

Denomination in Fig. S3	Bacterial strain	Growth on maltose minimal medium (MMM)^a^	Color of colonies on MacConkey maltose (MCM)^a^
−	GSJ101 pTorA-MalE, pHSG575	−	Pale
+	GSJ101 pTorA-MalE, pHSG-TatABC	+++	Red
1	GSJ101 pTorA-MalE, pHSG-TatA[N22pea]BC	−	Pale
2	GSJ101 pTorA-MalE, pHSG-TatA[N19pea]BC	−	Light red (pink)^b^
3	GSJ101 pTorA-MalE, pHSG-TatA[N17pea]BC	−	Pale
4	GSJ101 pTorA-MalE, pHSG-TatA[N16pea]BC	−	Light red (pink)^b^
5	GSJ101 pTorA-MalE, pHSG-TatA[N15pea]BC	−	Pale
6	GSJ101 pTorA-MalE, pHSG-TatA[N14pea]BC	+	Red (weak)
7	GSJ101 pTorA-MalE, pHSG-TatA[N11pea]BC	+++	Red
8	GSJ101 pTorA-MalE, pHSG-TatA[N10pea]BC	+++	Red
9	GSJ101 pTorA-MalE, pHSG-TatA[N8pea]BC	+++	Red
10	GSJ101 pTorA-MalE, pHSG-TatA[N7pea]BC	+++	Red

^a^Bacterial strains were streaked on minimal medium agar plates containing 0.4% maltose as the sole carbon source or on MacConkey agar plates containing 1% maltose and incubated at 37 °C. +  +  + , fast growth; + , slow growth; −, no growth.

^b^Color presumably due to partial cell lysis (see text for further details).

As expected, the presence of authentic *E. coli* TatA results in full activity of the TatABC translocase (Table 1 and Fig. S3), in line with published data^36, 42^. In contrast, chimeras ecoTatA[N22pea] and ecoTatA[N19pea], both carrying the entire TMH of pea TatA (Fig. 4A), are not able to substitute for *E. coli* TatA in the bacterial system, as indicated by the lack of growth of the corresponding strains on MMM (Table 1 and Fig. S3). The same holds true also for chimeras ecoTatA[N17pea], ecoTatA[N16pea], and ecoTatA[N15pea] in which the three C-terminal residues of pea TMH have successively been replaced. However, starting with chimera ecoTatA[N14pea], which shows low but definite TatA activity in the bacterial *in situ* plate assays, further reduction of the pea-derived residues in the chimeras allows significant Tat-dependent translocation of MalE into the periplasm of the corresponding *E. coli* strains. In almost all instances growth on MMM is accompanied by a colored phenotype of the corresponding colonies on MCM, while lack of growth on MMM corresponds to a pale colony on MCM (Table 1 and Fig. S3). However, in two cases (ecoTatA[N19pea] and ecoTatA[N16pea]) such a strict correlation is not observed. The corresponding colonies show a light-red or pink staining on MCM despite the fact that growth on MMM cannot be detected. The reason for this unusual phenotype is not known so far. We suggest that a fraction of the cells have undergone cell lysis upon expression of the respective TatA hybrid molecules thus resulting in the local acidification of the MCM medium to a level sufficient for the light red or pink phenotype exhibited by respective colonies. More importantly, however, the lack of growth on MMM of the ecoTatA[N19pea] or ecoTatA[N16pea]-expressing strains indicates that the corresponding chimeric TatA proteins do not allow Tat-dependent transport of the TorA-MalE reporter into the periplasm.

**Figure 7:**
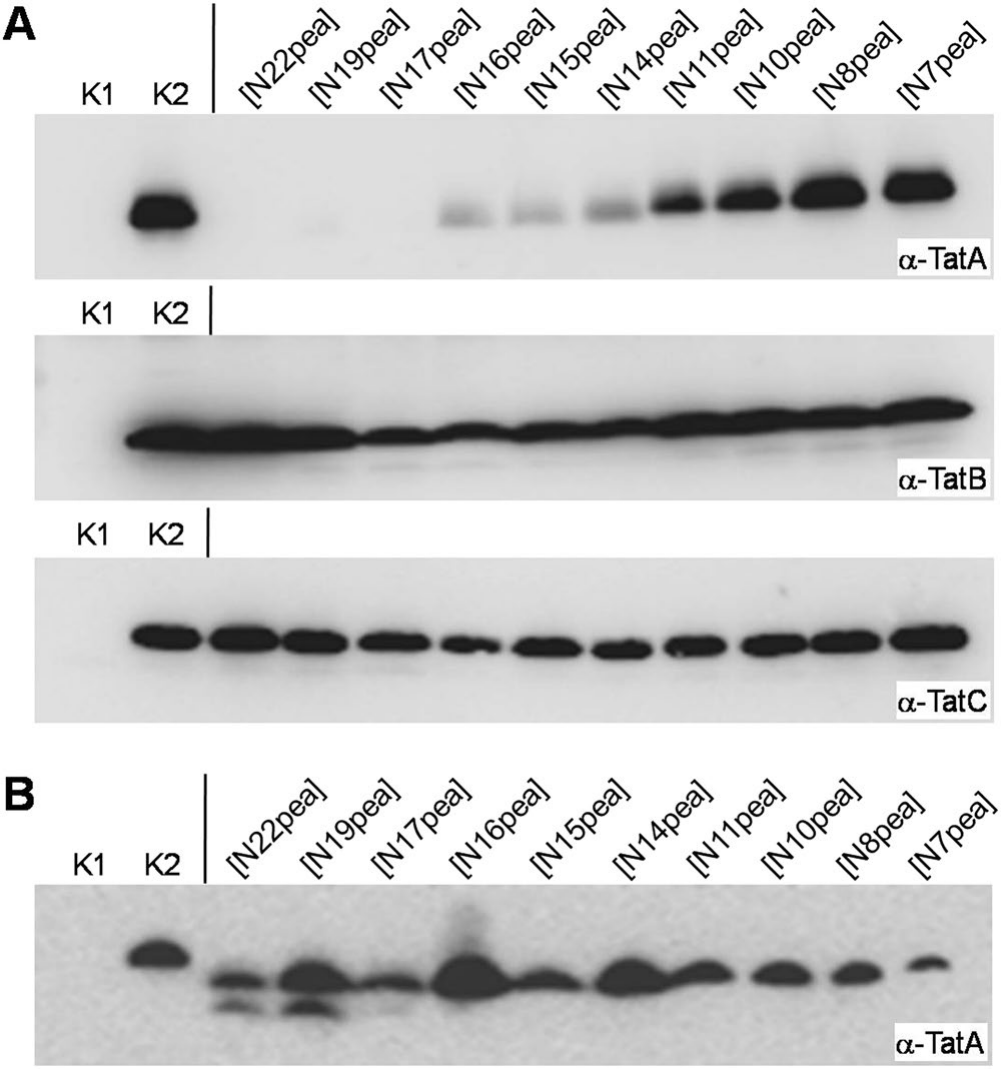
Accumulation of chimeric TatA proteins in *E. coli*. (**A**) Membrane preparations corresponding to identical amounts of cells were subjected to SDS-PAGE and immunoblotting using polyclonal antibodies raised against TatA (upper panel), TatB (middle panel), or TatC (lower panel) from *E. coli*. The immunoblots were developed by ECL-coupled detection. (**B**) Whole cell extracts of the cultures shown in (**A**) were analysed. K1, negative control (GSJ101 pTorAMalE, pHSG575); K2, positive control (GSJ101 pTorA-MalE, pHSG-TatABC). The other samples correspond to GSJ101 pTorA-MalE containing in addition pHSG-TatABC plasmids expressing the TatA chimeras indicated above the respective lanes.

Taken together, the activity of the chimeric TatA proteins in *E. coli* appears to be inversely correlated to that obtained for the same proteins in the thylakoid system, i.e. highly active chimeras in bacterial Tat transport are hardly active in thylakoidal Tat transport and *vice versa* (Table 1 and Fig. 5C). At first glance, this result seems to reinforce the presumed specific interaction of the transmembrane helix of TatA with the other components of the Tat machinery. However, for the bacterial system such a conclusion appears premature and superficial because we observed a striking correlation between the activities of the chimeric TatA proteins and their accumulation in the cytoplasmic membrane of *E. coli*. The inactive chimeras are not (ecoTatA[N22pea], ecoTatA[N19pea], ecoTatA[N17pea]) or at only low levels (ecoTatA[N16pea], ecoTatA[N15pea]) dectable in the membrane fractions of the respective strains when analysed by Western blotting using polyclonal antisera raised against *E. coli* TatA (Fig. 7A). However, all TatA chimeras are clearly detectable in whole cell extracts (Fig. 7B) indicating that they are all synthesized to considerable degrees. Hence, it is not yet possible to distinguish whether in the *E. coli* system transport activity of a given chimeric TatA protein reflects the extent of interaction with bacterial TatB and/or TatC, which might even be a prerequisite for stable membrane integration, or whether it is a direct consequence of the ability of chimeras to integrate into the cytoplasmic membrane of the respective bacterial reporter strain.

### Membrane interaction of chimeric TatA proteins in the thylakoid system

With thylakoid membranes, on the other hand, no such differences in membrane integration of the chimeric TatA proteins became apparent (Fig. S4). Still, we wanted to assess if active and inactive TatA proteins differ from each other with respect to persistance against membrane extraction. For this purpose, we have analysed *E. coli* TatA as well as five TatA chimeras (ecoTatA[N7pea], ecoTatA[N8pea], ecoTatA[N11pea], ecoTatA[N16pea], and ecoTatA[N17pea]) showing transport activities in the thylakoid system ranging from 0% to 100% (see Figs. 4 and 5). All proteins were obtained by *in vitro* translation in radiolabelled form and allowed to integrate into thylakoids under transport conditions. The thylakoids were subsequently treated with either (i) HM buffer, (ii) 0.2 M Na_2_CO_3_, (iii) 0.2 M NaBr, (iv) 0.6 M NaBr, or (v) 1 M urea. Membrane and supernatant fractions were separated by centrifugation and analysed by SDS-PAGE and phosphorimaging.

**Figure 8:**
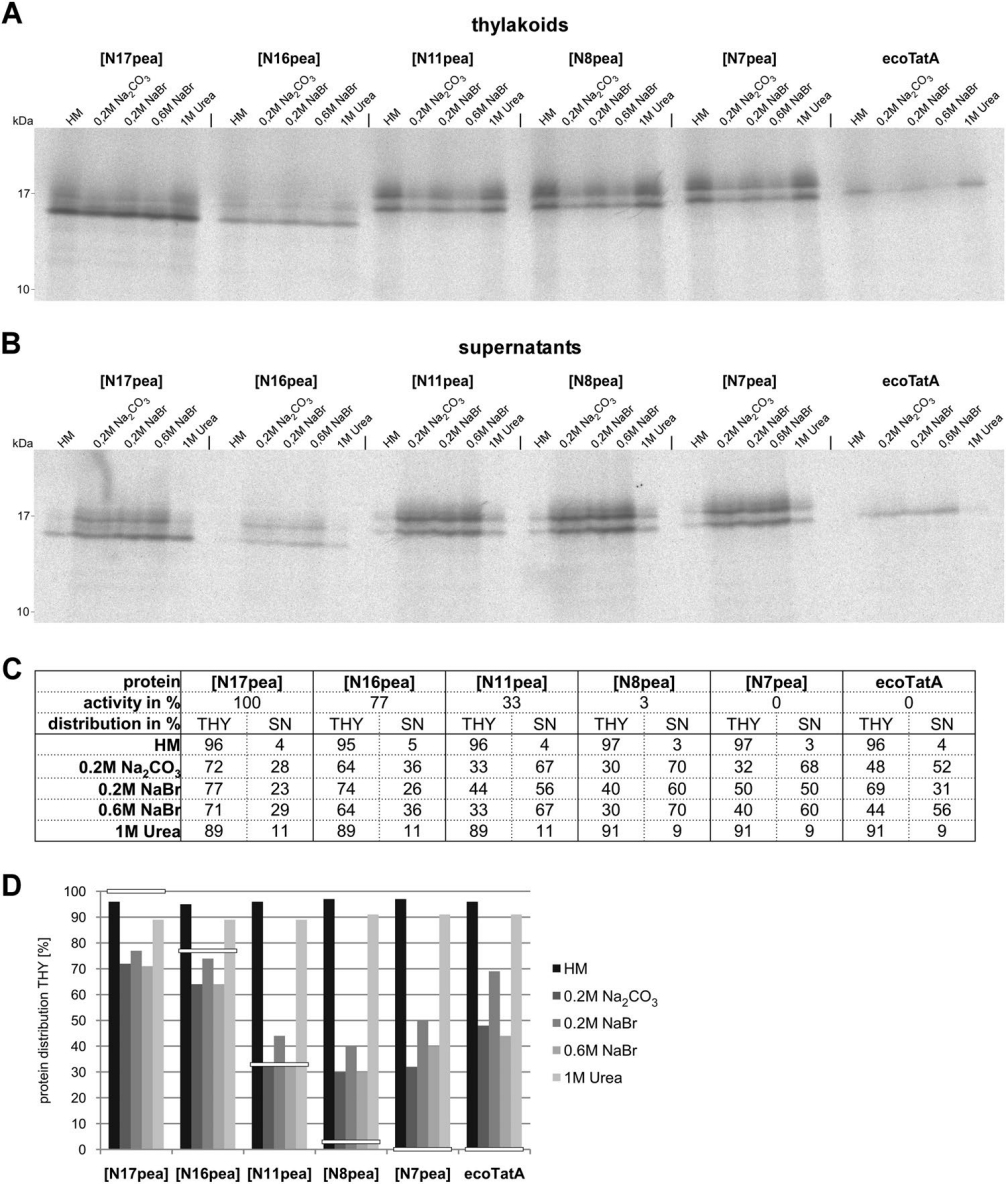
Integration of functional and non-functional TatA chimeras in the thylakoid membrane. *E. coli* TatA as well as five TatA chimeras (ecoTatA[N7pea], ecoTatA[N8pea], ecoTatA[N11pea], ecoTatA[N16pea], and ecoTatA[N17pea]) which show transport activities in the thylakoid system ranging from 0% to 100% (see Figs. 4 and 5) were obtained by *in vitro* translation in radiolabelled form and incubated with thylakoid vesicles under transport conditions for 15 min at 25 °C in the light. Thylakoids were washed once with HM buffer and subsequently resuspended in either (i) *HM buffer*, (ii) *0.2 M Na*_*2*_ *CO*_*3*_, (iii) *0.2 M NaBr*, (iv) *0.6 M NaBr*, or (v) *1 M Urea*. After incubation for 30 min on ice thylakoids (**A**) and supernatant fractions (**B**) were collected after centrifugation and analysed by SDS-PAGE and phosphorimaging. The relative amounts of each TatA protein in the two fractions *(THY* and *SN*, respectively) were quantified (**C**). (**D**) Graph showing the relative amounts of each TatA protein in the THY fractions after membrane extraction. The activities of the different TatA proteins in thylakoid transport experiments are indicated by *white bars*.

It turned out that all proteins analysed show considerable membrane binding and integration properties, irrespective of their activity in thylakoid transport experiments (Fig. 8). They are all resistant against membrane extraction with HM buffer or 1 M urea demonstrating that they had been properly inserted into the thylakoids. Treatment with solutions of NaBr or Na_2_CO_3_ reveals some differences between the chimeras though. EcoTatA[N17pea] and ecoTatA[N16pea], which are 100% and 77% active in the thylakoid assays, respectively, show membrane persistance rates ranging from 64% to 77% (Fig. 8C). In contrast, ecoTatA[N11pea], which has 33% activity in thylakoid transport, is retained to only 33–44% in the membrane. At first glance, this seems to suggest a certain degree of correlation between membrane persistence and catalytic activity. However, the same or even higher resistance against membrane extraction by NaBr or Na_2_CO_3_ is found for ecoTatA[N7pea], ecoTatA[N8pea], and even *E. coli* TatA (Fig. 8C,D) despite the fact that the three proteins are barely (ecoTatA[N8pea], Fig. 5) or not at all active in the thylakoid system (Figs. 2 and 5).

From these results we conclude that in the thylakoid system the catalytic transport activity of a given TatA protein is not a simple consequence of its extent of membrane integration but rests instead to a larger degree on processes taking place after membrane binding.

## Discussion

It was the goal of this study to find out if chimeric TatA proteins composed of segments derived from both, *E. coli* TatA and pea TatA, can principally replace the respective authentic TatA proteins in bacterial and plant Tat transport systems and, if so, to determine which residues of either of the original TatAs are required to render the chimeric proteins catalytically active in the respective heterologous system.

### The TMH of TatA determines its activity in thylakoidal Tat transport

Starting from the observation that plant and bacterial TatA proteins share substantial sequence and structure homology (Figs 1 and S2) it was surprising that *E. coli* TatA is not able to replace pea TatA in *in thylakoido* transport assays (Fig. 2). Such assays have been previously shown to allow determination of transport activity of heterologous plant TatA proteins^29^ and should, in principle, be suitable for any given TatA. The lack of bacterial TatA activity observed prompted us to generate a large set of chimeric TatA proteins in order to potentially identify the underlying cause for this incapability. It turned out that it is exclusively the N-terminal region comprising the transmembrane helix which determines the activity of TatA in thylakoidal Tat transport since all other parts of the pea protein can be replaced by their bacterial counterparts without notable effect (Fig. 4). Further, the substitution of just three amino acid residues of *E. coli* TatA (S5, W7, Q8) by the corresponding residues from pea TatA (G, P, and E, respectively) is sufficient to achieve at least minimal catalytic activity in our thylakoid transport assays (Fig. 6). This is in line with published data demonstrating that both, Q8 of *E. coli* TatA and the corresponding E10 residue of pea TatA, are indispensable for TatA function in the respective transport systems^29, 35, 44^. Taken together, these results demonstrate that the two TatA proteins, and hence probably the entire Tat machinery, are structurally and functionally closely related to each other despite their enormous evolutionary distance.

Except for the three essential residues described above, no further explicit key residues determining the catalytic activity could be detected. Instead, it appears that all residues in the transmembrane helix are involved in the transport process, in yet an unknown, sequence-specific manner. This is evident in that almost each substitution of a pea residue within TMH by its bacterial counterpart leads to a decrease in catalytic activity in the *in thylakoido* assays (Fig. 5). In contrast, the very N-terminal residues, which differ between pea and *E. coli* in both sequence and length (Fig. 1), apparently do not play a major role, since corresponding mutant pairs carrying or lacking these residues (e.g., ecoTatA[N11pea] and ecoTatA[4–11pea], respectively) show similar catalytic activities (Figs 5C and 6C).

### TatA chimeras show reciprocal activities in the two assay systems

These findings are in principle valid also for the complementary, bacterial system. If transport activity of the chimeric TatA proteins is determined in *E. coli*, basically reciprocal results to those achieved in the thylakoid system are obtained, i.e. highly active chimeras in thylakoidal Tat transport are merely active in bacterial Tat transport and *vice versa* (compare Table 1 with Figs 4C and 5C). At first glance this seems to suggest that Tat translocation depends on specific interaction of the TMH of TatA with the other components of the Tat machinery and that bacterial and thylakoidal Tat components have cumulated deviating and probably compensating mutations within the protein interacting segments which prevent mutual exchange of single subunits of the translocases.

However, this interpretation is compromised by the fact that the TatA derivatives analysed here are not found in equal amounts in the bacterial membranes. In fact, those chimeras lacking transport activity in *E. coli* are detected, if at all, only at low levels in the respective membrane fractions by immunoblotting with antisera against *E. coli* TatA, while detectable transport activity is accompanied by somewhat stronger accumulation of the protein in the bacterial membranes (Table 1 and Fig. 7). This seemingly points to a rather simple, direct correlation of protein accumulation in the membrane and transport activity. However, it clearly contradicts the results obtained in the thylakoid system which do not show such strict correlation. Both active and inactive TatA chimeras are resistant to membrane extraction with 1 M urea (Fig. 8). Upon extraction with solutions of chaotropic salts or alkaline pH highly active TatA chimeras (≥77% activity) show somewhat stronger membrane persistance than chimeras with lower (33%) or no catalytic activity. However, the differences are too small and too inconsistent (Fig. 8D) to support the assumption that transport activity of a given TatA chimera in the thylakoid system is determined to a large extent by its membrane integration characteristics.

Hence, it appears likely that the reasons for lack of transport activity of the chimeric TatA proteins are different for the two analytical systems, although it cannot even be ruled out that lack of membrane accumulation of several TatA chimeras in *E. coli* is actually a consequence of their lacking transport activity. It is, for example, well conceivable that impairment in the productive interaction of a given TatA chimera with bacterial TatB and/or TatC leads in turn to its instability in the membrane. However, formal proof for such kind of speculation is missing.

### Is the activity of TatA correlated with the hydrophobicity of its TMH?

The finding that the entire transmembrane domain of TatA has an apparent influence on the transport activity of the chimeric TatA proteins can be interpreted in two ways. Either, there are numerous residues along the TMH which are involved in specific interaction with TatB and/or TatC, or a more general physico-chemical attribute of this domain is responsible for the observed effect. In fact, closer inspection of the TatA chimeras with regard to their transport activity in the thylakoid system and amino acid composition within TMH show a remarkable interrelation. Due to the overall stronger hydrophobicity of the transmembrane helix of *E. coli* TatA compared to that of pea (Fig. S5), almost all substitutions within pea TMH lead to an increase in hydrophobicity. In most instances this is accompanied by a considerable decrease in transport activity in the thylakoid system (Figs 5 and 6). This holds true for each of the substitutions A17V, A16V, G14V, V11I, and V10L (Table 2). In the case of V10L the effect appears weaker at first sight (from 9% to 3%) but even this actually represents a tripartition of transport activity. The only exception of this presumed rule is V15I, which shows no major impact on thylakoidal transport activity despite increased hydrophobicity (Fig. 5 and Table 2).

**Table 2:** Increasing hydrophobicity of TMH affects TatA activity in the thylakoid system.

ecoTatA[…]	mutation	activity in %
N17pea		100
▼	A17V	
N16pea		77
▼	A16V	
N15pea		55
▼	V15I	
N14pea		53
▼	G14V	
N11pea		33
▼	V11I	
N10pea		9
▼	V10L	
N8pea		3

How could such relatively mild changes in hydrophobicity by single site mutations have such strong and reciprocal effects on transport activity of TatA in the two systems? One obvious possibility is the lipid composition which indeed differs remarkably between the bacterial and thylakoidal membranes. While the cytoplasmic membrane of *E. coli* contains predominantly phospholipids, particularly phosphatidylethanolamine^45^, the thylakoid membrane consists instead to more than 70% of galactolipid derivatives, namely monogalactosyl diacylglycerol (MGDG, approx. 50%) and digalactosyl diacylglycerol (DGDG, >20%)^40^. Although the potential role of such divergent lipid composition on the activity of protein transport machineries has not been addressed so far, an influence on the assembly of the Tat components analogous to that described in the structural organisation of the light-harvesting complex of photosystem II^46^ appears possible.

A second point to be considered is the observation that in chloroplasts TatA is also present in the hydrophilic stromal compartment^33^. Furthermore, in *in thylakoido* reconstitution assays such soluble TatA can fully replace membrane-bound TatA that was inactivated by antibody treatment^29, 35^. Despite the fact that the protein is known to fulfil its function in membrane-bound form, a temporary, and potentially obligatory, stage of soluble TatA therefore appears possible, at least in chloroplasts. In such a scenario, increasing hydrophobicity of TMH of TatA would probably decrease the solubility of TatA in the stroma and thus impair transport activity.

## Materials and Methods

### Cloning and mutagenesis

The DNA fragments comprising the entire reading frames of either TatA from *E. coli* or mature TatA from pea were amplified by PCR using primer pairs EcoTatA forward + EcoTatA reverse and peaTatA forward + peaTatA reverse, respectively (Table S1). After restriction with NcoI and SmaI, both PCR products were cloned separately with vector pIVEX 1.3 WG that was linearised accordingly. The two clones were subsequently used as templates for PCR reactions using primer pairs ecoTatA[N22pea] TMH + HR (forward + reverse) and ecoTatA[N22pea] APH + CTD (forward + reverse), respectively (Table S1), which amplify complementing halfs of vector pIVEX 1.3 plus either the coding region of TMH and HR from pea TatA or the coding region of APH and CTD from *E. coli* TatA, respectively (Fig. 4A). Both PCR fragments were cleaved with EcoRI and ligated as EcoRI/blunt end fragments yielding clone ecoTatA[N22pea] in vector pIVEX 1.3 (Fig. 4A), which was taken as source for TatA derivatives from ecoTatA[N19pea] to ecoTatA[N7pea]. In contrast,TatA derivatives from ecoTatA[8pea] to ecoTatA[4–14pea] were generated using clone ecoTatA as template. The actual mutagenesis reactions were carried out with the QuikChange® Site-Directed Mutagenesis Kit (Stratagene, La Jolla, CA, USA) using the primers listed in Table S1 and confirmed by DNA sequencing.

For the construction of plasmid pHSG-TatABC, a *Bam*HI/*Pst*I fragment containing the *E. coli tatA*, *tatB*, and *tatC* genes was isolated from plasmid pHSG-TatABCE^36^ and ligated into *Bam*HI/*Pst*I-digested pHSG575^47^. To replace the *E. coli tatA* gene in plasmid pHSG-TatABC by the genes encoding the chimeric TatA proteins N22pea, N19pea, N17pea, N16pea, N15pea, N14pea, N11pea, N10pea, N8pea, or N7pea, respectively, the corresponding *tatA* genes were amplified in a PCR reaction using primers AE FW BamHI and AE Rev EcoRV (Table S1) and the respective source clones in vector pIVEX 1.3 WG (see above) as templates. The resulting PCR fragments were digested with *Bam*HI and *Eco*RV and ligated together with the larger of the two DNA fragments that were obtained by digesting pHSG-TatABC with the same two enzymes.

### In thylakoido protein transport and membrane binding experiments

Isolation of chloroplasts and thylakoids from pea seedlings (*P. sativum* var. Feltham First) was carried out according to^48^. Thylakoid vesicles corresponding to 15 μg of chlorophyll were incubated with radiolabelled precursor protein obtained by *in vitro* transcription/translation according to^12^ in the presence of [^35^S]-methionine. After incubation for 15 min at 25 °C in the light the assays were diluted with one volume of HM buffer (10 mM HEPES/KOH pH 8,0; 5 mM MgCl_2_) and thylakoids were recovered by centrifugation (4 min at 20,000 g). Pellets were washed once, resuspended in HM buffer and divided into two aliquots corresponding to 7.5 μg of chlorophyll each. One aliquot was treated with thermolysin (182 μg/ml) for 30 min on ice, the other aliquot was mock treated. Proteolysis was stopped by the addition of one volume HM buffer supplemented with 10 mM EDTA. Thylakoids were recovered by centrifugation and analysed on 10–17.5% SDS-polyacrylamide gradient gels followed by phosphorimaging.

Modifications, like anti-TatA treatment of thylakoids and supplementation of the assays with soluble TatA proteins obtained from *in vitro* translation with the wheat germ rapid translation system (*RTS*), were carried out following the protocols of^29, 49^.

Membrane binding and integration experiments were performed under *in thylakoido* transport conditions with radiolabelled TatA proteins obtained from *in vitro* translation with the RTS system. After incubation, thylakoid vesicles were washed once with HM buffer and divided into aliquots corresponding to 7.5 μg of chlorophyll each. Thylakoids of each aliquot were resuspended with HM buffer supplemented with either 0.2 M Na_2_CO_3_, 0.2 M NaBr, 0.6 M NaBr, or 1 M urea and incubated for 30 min on ice. Thylakoids and supernatant fractions were collected after centrifugation (4 min at 20,000 g) and analysed by SDS-PAGE and phosphorimaging. The gels were analysed with the Fujifilm FLA-3000 (Fujifilm, Düsseldorf, Germany) utilising the software packages BAS-Reader (version 3.14) and AIDA (version 3.25; Raytest, Straubenhardt, Germany) which was used also for quantification of the data.

### *E. coli* methods

*E. coli* strains XL1 Blue (Stratagene) and GSJ101^36^ were grown at 37 °C in LB medium^50^, minimal medium^51^ supplemented with 0.4% maltose, or MacConkey agar base medium (Difco) supplemented with 1% maltose. If required, isopropyl-ß-d-thiogalactopyranoside (IPTG) was used in a 0.1 mM concentration. Antibiotic supplements were used in the following concentrations: kanamycin, 50 mg/l; chloramphenicol, 25 mg/l. For the preparation of *E. coli* whole cell extracts, 5 ml of an overnight culture were centrifuged for 10 min at 18,320 g. The resulting cell pellet was washed once with 0.9% NaCl and resuspended in 200 μl 50 mM Tris-HCl pH 8.0. After adding 50 μl 5x sample buffer (250 mM Tris-HCl, pH 6.8, 10% SDS, 30% (v/v) glycerol, 10 mM dithiothreitol, 0.05% (w/v) bromophenol blue) the samples were incubated for 15 min at 95 °C and subjected to SDS-PAGE. Preparation of membranes and Western blotting was performed as described^43^. The antibodies against TatA, TatB, and TatC that were used in this study were raised in rabbits by Eurogentec (Liège, Belgium) against two synthetic peptides from each of the respective Tat components. The peptides used to generate the TatA-specific antibodies were TatA1 (amino acids 55–70: QDADFTAKTIADKQAD) and TatA2 (amino acids 74–89: EQAKTEDAKRHDKEQV). The peptides used to generate the TatB-specific antibodies were TatB1 (amino acids 69–84: ASLTNLTPELKASMDE) and TatB2 (amino acids 156–171: AEPKTAAPSPSSSDKP). The peptides used to generate the TatC-specific antibodies were TatC1 (amino acids 1–15: MSVEDTQPLITHLIE) and TatC2 (amino acids 243–258: REEENDAEAESEKTEE).

### Miscellaneous

Radiolabelled proteins were subjected to gel electrophoresis under denaturing conditions as described by^52^. All other methods followed published protocols^53^.

## Electronic supplementary material

Supplementary Information 

## References

[1] Fröbel, J., Rose, P. & Müller, M. Twin-arginine-dependent translocation of folded proteins. *Philos. Trans. R. Soc. Lond. B Biol. Sci*., 1029–1046 (2012). 10.1098/rstb.2011.0202 PMC3297433 22411976

[2] Patel R, Smith SM, Robinson C (2014). Protein transport by the bacterial Tat pathway. Biochim. Biophys. Acta.

[3] Berks BC (2015). The twin-arginine protein translocation pathway. Annu. Rev. Biochem..

[4] Cline K (2015). Mechanistic Aspects of Folded Protein Transport by the Twin Arginine Translocase (Tat). J. Biol. Chem..

[5] Chaddock AM (1995). A new type of signal peptide: central role of a twin-arginine motif in transfer signals for the delta pH-dependent thylakoidal protein translocase. EMBO J..

[6] Berks BC (1996). A common export pathway for proteins binding complex redox cofactors?. Mol. Microbiol..

[7] Bageshwar UK, Musser SM (2007). Two electrical potential-dependent steps are required for transport by the *Escherichia coli* Tat machinery. J. Cell Biol..

[8] Braun NA, Davis AW, Theg SM (2007). The chloroplast Tat pathway utilizes the transmembrane electric potential as an energy source. Biophys. J..

[9] Clark SA, Theg SM (1997). A folded protein can be transported across the chloroplast envelope and thylakoid membranes. Mol. Biol. Cell..

[10] Hynds PJ, Robinson D, Robinson C (1998). The Sec-independent twin-arginine-translocation system can transport both tightly folded and malfolded proteins across the thylakoid membrane. J. Biol. Chem..

[11] Sargent F (1998). Overlapping functions of components of a bacterial Sec-independent protein export pathway. EMBO J..

[12] Marques JP, Dudeck I, Klösgen RB (2003). Targeting of EGFP chimeras within chloroplasts. Mol. Genet. Genomics.

[13] Santini CL (1998). A novel sec-independent periplasmic protein translocation pathway in *Escherichia coli*. EMBO J..

[14] Molik S, Karnauchov I, Weidlich C, Herrmann RG, Klösgen RB (2001). The Rieske Fe/S protein of the cytochrome *b*_6_*/f*-complex: missing link in the evolution of protein transport pathways in chloroplasts?. J. Biol. Chem..

[15] Brüser T, Yano T, Brune DC, Daldal F (2003). Membrane targeting of a folded and cofactor-containing protein. Eur. J. Biochem..

[16] Müller M, Klösgen RB (2005). The Tat pathway in bacteria and chloroplasts. Mol. Membrane Biol..

[17] Rollauer SE (2012). Structure of the TatC core of the twin-arginine protein transport system. Nature.

[18] Koch S, Fritsch MJ, Buchanan G, Palmer T (2012). *Escherichia coli* TatA and TatB proteins have N-out, C-in topology in intact cells. J. Biol. Chem..

[19] Cline K, Mori H (2001). Thylakoid ΔpH-dependent precursor proteins bind to a cpTatC–Hcf106 complex before Tha4-dependent transport. J. Cell Biol..

[20] Alami M (2003). Differential Interactions between a Twin-Arginine Signal Peptide and Its Translocase in *Escherichia coli*. Mol. Cell.

[21] Richter S, Brüser T (2005). Targeting of unfolded PhoA to the TAT translocon of *Escherichia coli*. J. Biol. Chem..

[22] Mori H, Cline K (2002). A twin arginine signal peptide and the pH gradient trigger reversible assembly of the thylakoid [Delta]pH/Tat translocase. J. Cell Biol..

[23] Rodriguez F (2013). Structural model for the protein-translocating element of the twin-arginine transport system. Proc. Natl. Acad. Sci. USA.

[24] Berghöfer J, Klösgen RB (1999). Two distinct translocation intermediates can be distinguished during protein transport by the TAT (Deltaph) pathway across the thylakoid membrane. FEBS Lett..

[25] Jakob M, Kaiser S, Gutensohn M, Hanner P, Klösgen RB (2009). Tat subunit stoichiometry in Arabidopsis thaliana challenges the proposed function of TatA as the translocation pore. Biochim. Biophys. Acta.

[26] Gohlke U (2005). The TatA component of the twin-arginine protein transport system forms channel complexes of variable diameter. Proc. Natl. Acad. Sci. USA.

[27] Cline K, McCaffery M (2007). Evidence for a dynamic and transient pathway through the TAT protein transport machinery. EMBO J..

[28] Natale P, Brüser T, Driessen AJ (2008). Sec- and Tat-mediated protein secretion across the bacterial cytoplasmic membrane - distinct translocases and mechanisms. Biochim. Biophys. Acta.

[29] Hauer RS (2013). Enough is enough: TatA demand during Tat-dependent protein transport. Biochim. Biophys. Acta.

[30] Jack RL, Sargent F, Berks BC, Sawers G, Palmer T (2001). Constitutive expression of *Escherichia coli tat* genes indicates an important role for the twin-arginine translocase during aerobic and anaerobic growth. J. Bacteriol..

[31] Mori H, Summer EJ, Cline K (2001). Chloroplast TatC plays a direct role in thylakoid (Delta)pH-dependent protein transport. FEBS Lett..

[32] Celedon JM, Cline K (2012). Stoichiometry for binding and transport by the twin arginine translocation system. J. Cell. Biol..

[33] Frielingsdorf S, Jakob M, Klösgen RB (2008). A stromal pool of TatA promotes Tat-dependent protein transport across the thylakoid membrane. J. Biol. Chem..

[34] Pop OI (2003). Sequence-specific binding of prePhoD to soluble TatAd indicates protein-mediated targeting of the Tat export in *Bacillus subtilis*. J. Biol. Chem..

[35] Dabney-Smith C, Mori H, Cline K (2003). Requirement of a Tha4-conserved transmembrane glutamate in thylakoid Tat translocase assembly revealed by biochemical complementation. J. Biol. Chem..

[36] Blaudeck N, Kreutzenbeck P, Freudl R, Sprenger GA (2003). Genetic analysis of pathway specificity during posttranslational protein translocation across the *Escherichia coli* plasma membrane. J. Bacteriol..

[37] Zoufaly S (2012). Mapping precursor-binding site on TatC subunit of twin arginine-specific protein translocase by site-specific photo cross-linking. J. Biol. Chem..

[38] Wexler M (1998). Targeting signals for a bacterial Sec-independent export system direct plant thylakoid import by the ΔpH pathway. FEBS Lett..

[39] Halbig D, Hou B, Freudl R, Sprenger GA, Klösgen RB (1999). Bacterial proteins carrying twin-R signal peptides are specifically targeted by the ΔpH-dependent transport machinery of the thylakoid membrane system. FEBS Lett..

[40] Sprague SG, Staehelin LA (1984). A rapid reverse phase evaporation method for the reconstitution of uncharged thylakoid membrane lipids that resist hydration. Plant Physiol..

[41] Hicks MG (2003). The *Escherichia coli* twin-arginine translocase: conserved residues of TatA and TatB family components involved in protein transport. FEBS Lett..

[42] Lausberg F (2012). Genetic evidence for a tight cooperation of TatB and TatC during productive recognition of twin-arginine (Tat) signal peptides in *Escherichia coli*. PLoS One.

[43] Kreutzenbeck P (2007). *Escherichia coli* twin arginine (Tat) mutant translocases possessing relaxed signal peptide recognition specificities. J. Biol. Chem..

[44] Greene NP (2007). Cysteine Scanning Mutagenesis and Disulfide Mapping Studies of the TatA Component of the Bacterial Twin Arginine Translocase. J. Biol. Chem..

[45] Cronan JE (2003). Bacterial membrane lipids: where do we stand?. Annu. Rev. Microbiol..

[46] Schaller S (2011). Regulation of LHCII aggregation by different thylakoid membrane lipids. Biochim. Biophys. Acta (BBA) - Bioenergetics.

[47] Takeshita S, Sato M, Toba M, Masahashi W, Hashimoto-Gotoh T (1987). High-copy-number and low-copy-number plasmid vectors for *lacZα*-complementation and chloramphenicol- or kanamycin-resistance selection. Gene.

[48] Hou B, Frielingsdorf S, Klösgen RB (2006). Unassisted membrane insertion as the initial step in DeltapH/Tat-dependent protein transport. J. Mol. Biol..

[49] Dittmar J, Schlesier R, Klösgen RB (2014). Tat transport of a Sec passenger leads to both completely translocated as well as membrane-arrested passenger proteins. BBA - Molecular Cell Research.

[50] Miller, J. H. A Short Course in Bacterial Genetics: A Laboratory Manual and Handbook for *Escherichia coli* and related Bacteria, Cold Spring Harbor Laboratory, Cold Spring Harbor, NY (1992).

[51] Tanaka H, Lerner SA, Lin ECC (1967). Replacement of a phosphoenolpyruvate-dependent phosphotransferase by a nicotinamide adenine dinucleotide-linked dehydrogenase for the utilization of mannitol. J. Bacteriol..

[52] Laemmli UK (1970). Cleavage of structural proteins during the assembly of the head of bacteriophage T4. Nature.

[53] Sambrook, J. & Russell, D. W. Molecular Cloning: A Laboratory manual. 3rd Edition. Cold Spring Harbor, New York: Cold spring Harbor Laboratories (2001).

[54] Huang X, Miller W (1991). A time-efficient, linear-space local similarity algorithm. Adv. Appl. Math..

[55] Schlesier R, Klösgen RB (2010). Twin arginine translocation (Tat)-dependent protein transport: the passenger protein participates in the initial membrane binding step. Biol. Chem..

