# Supplementary material for: How to achieve Tat transport with alien TatA
Source: Sci Rep. 2017 Aug 18;7:8808. doi: 10.1038/s41598-017-08818-w (PMC5562801; doi:10.1038/s41598-017-08818-w)
Supplement: Supplementary file 1 — Supplementary Information [file 41598_2017_8818_MOESM1_ESM.doc]

**Supplementary Material**

**How to achieve Tat transport with alien TatA**

René Steffen Hauer^1^, Roland Freudl^2^, Julia Dittmar^1^, Mario Jakob^1^, and Ralf Bernd Klösgen^1^*

^1^ Institute of Biology – Plant Physiology, MartinLutherUniversity Halle-Wittenberg,

06099 Halle/Saale, Germany

^2^ Institut für Bio- und Geowissenschaften 1, Biotechnologie, Forschungszentrum Jülich GmbH, 52425 Jülich, Germany

**
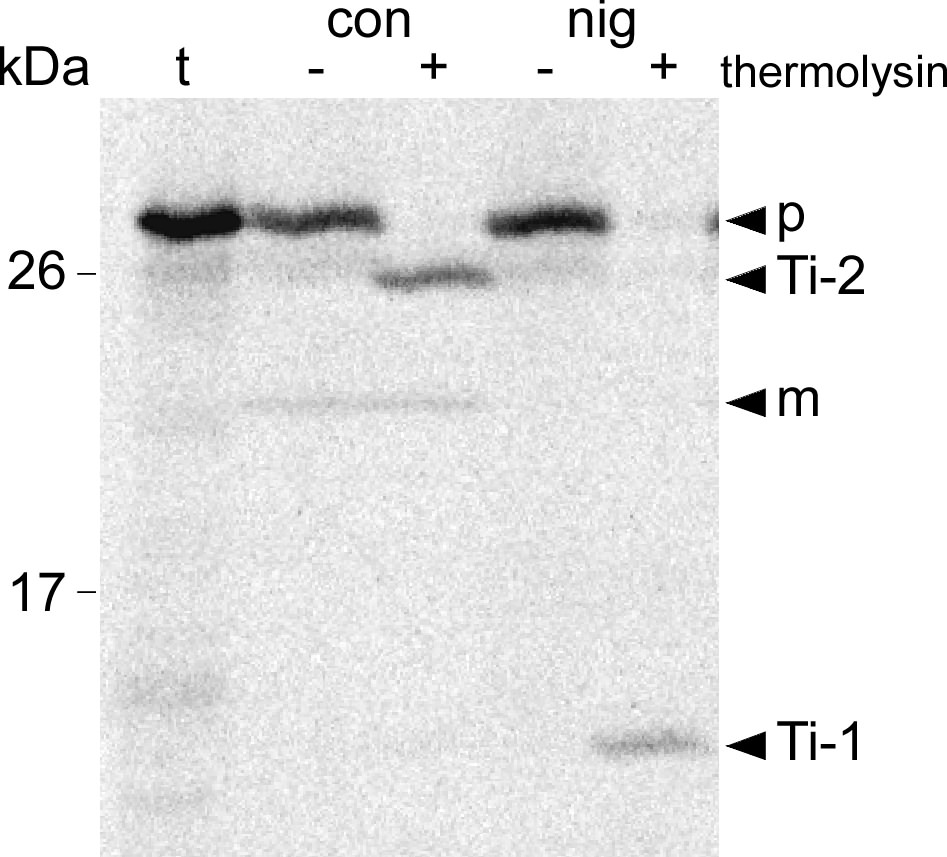
**

**Figure S1** *Tat-dependent protein transport depends on the transthylakoidal proton gradient.*

The chimeric precursor protein 16/23 was incubated with isolated pea thylakoids for 10 min at 25°C in the dark in the absence (*con*) or presence of 2 μM nigericin (*nig*), which dissipates the transthylakodial proton gradient. After washing with HM buffer the assays were divided and either treated with thermolysin *(+)* or mock-treated *(–)* For further details see the legend to Fig. 2.

In the presence of the transthylakoidal proton gradient both the mature 23 kDa protein and translocation intermediate Ti-2 can be observed which represents the Tat substrate after complete membrane translocation of the passenger polypeptide before removal of the transport signal^24,48^. If the proton gradient is dissipated by nigericin, solely the early translocation intermediate Ti-1 is found which represents the membrane-bound Tat substrate prior to translocation of the passenger protein^55^.


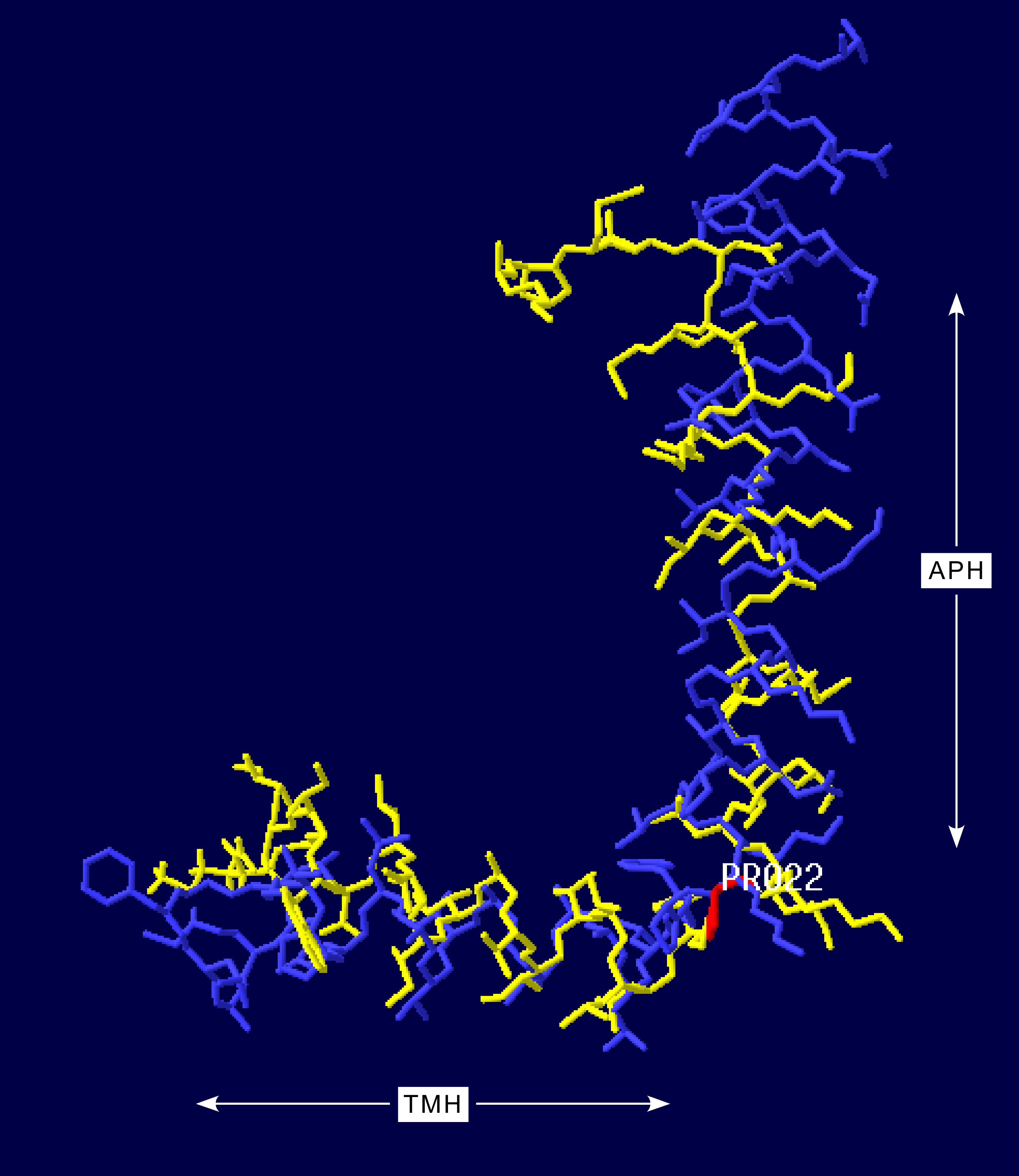
**Figure S2** *Putative structural conservation of TatA proteins from plants and bacteria.*

Overlay of a potential structure of TatA from *Arabidopsis thaliana* (*blue line*) modelled to one of the NMR structures deposited in the RCSB database (http://rcsb.org) for the T22P derivative of *E. coli* TatA^23^ (*yellow line*). The transmembrane helix (TMH) and the amphipathic helix (APH) of *E. coli* TatA are indicated. The proline residue of the T22P derivative is depicted in *red*.


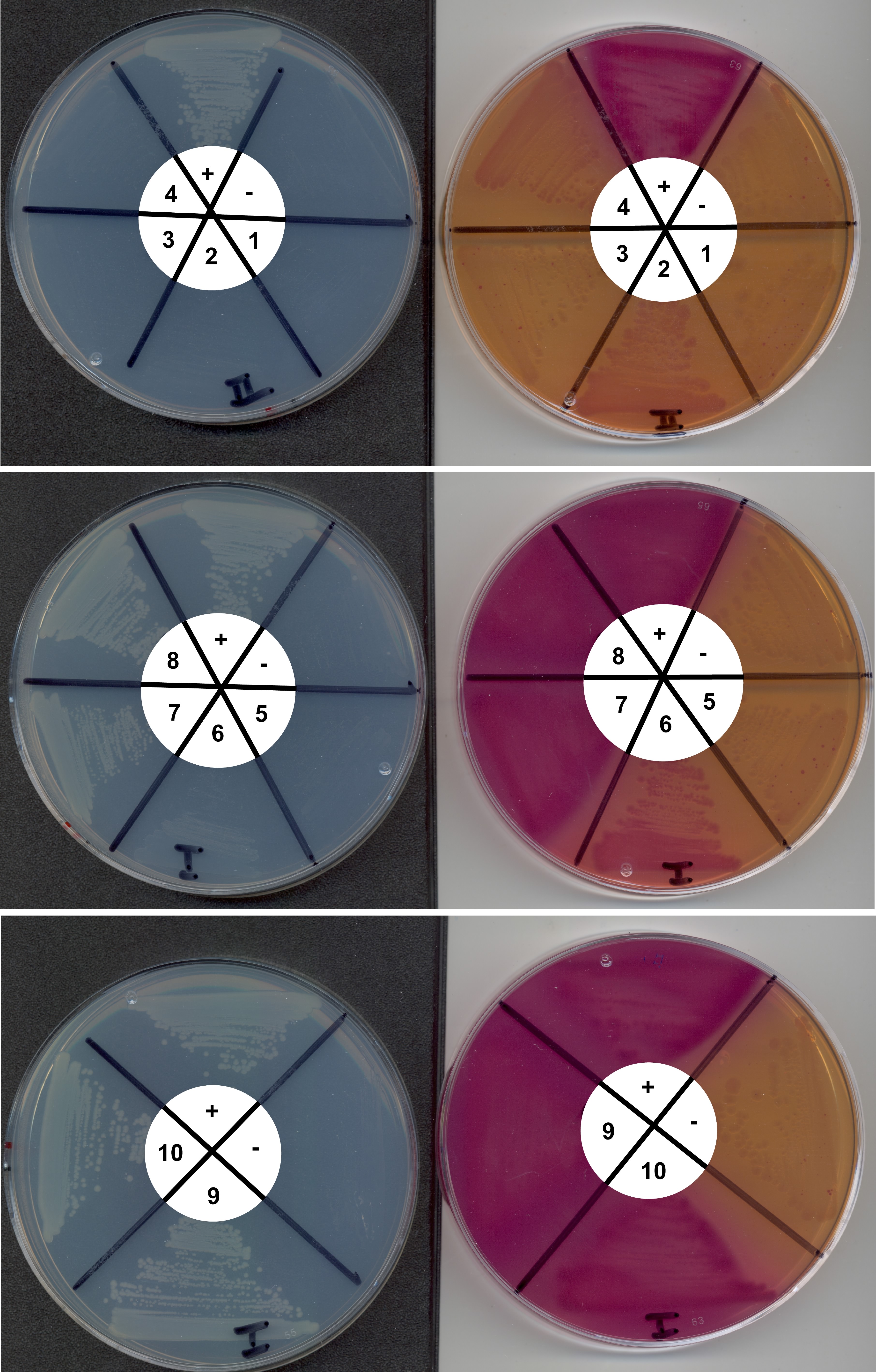
**Figure S3** *Phenotype of bacterial strains on maltose minimal medium and MacConkey maltose*

Bacterial strains were streaked on minimal medium agar plates containing 0.4% maltose as the sole carbon source (*left panel*) or on MacConkey agar plates containing 1% maltose (*right panel*) and incubated at 37°C. Assignment of the strains: *(–)* GSJ101 pTorA-MalE, pHSG575; *(+)* GSJ101 pTorA-MalE, pHSG-TatABC; *(1)* GSJ101 pTorA-MalE, pHSG-TatA[N22pea]BC; *(2)* GSJ101 pTorA-MalE, pHSG-TatA[N19pea]BC; *(3)* GSJ101 pTorA-MalE, pHSG-TatA[N17pea]BC; *(4)* GSJ101 pTorA-MalE, pHSG-TatA[N16pea]BC; *(5)* GSJ101 pTorA-MalE, pHSG-TatA[N15pea]BC; *(6)* GSJ101 pTorA-MalE, pHSG-TatA[N14pea]BC; *(7)* GSJ101 pTorA-MalE, pHSG-TatA[N11pea]BC; *(8)* GSJ101 pTorA-MalE, pHSG-TatA[N10pea]BC; *(9)* GSJ101 pTorA-MalE, pHSG-TatA[N8pea]BC; *(10)* GSJ101 pTorA-MalE, pHSG-TatA[N7pea]BC.

**
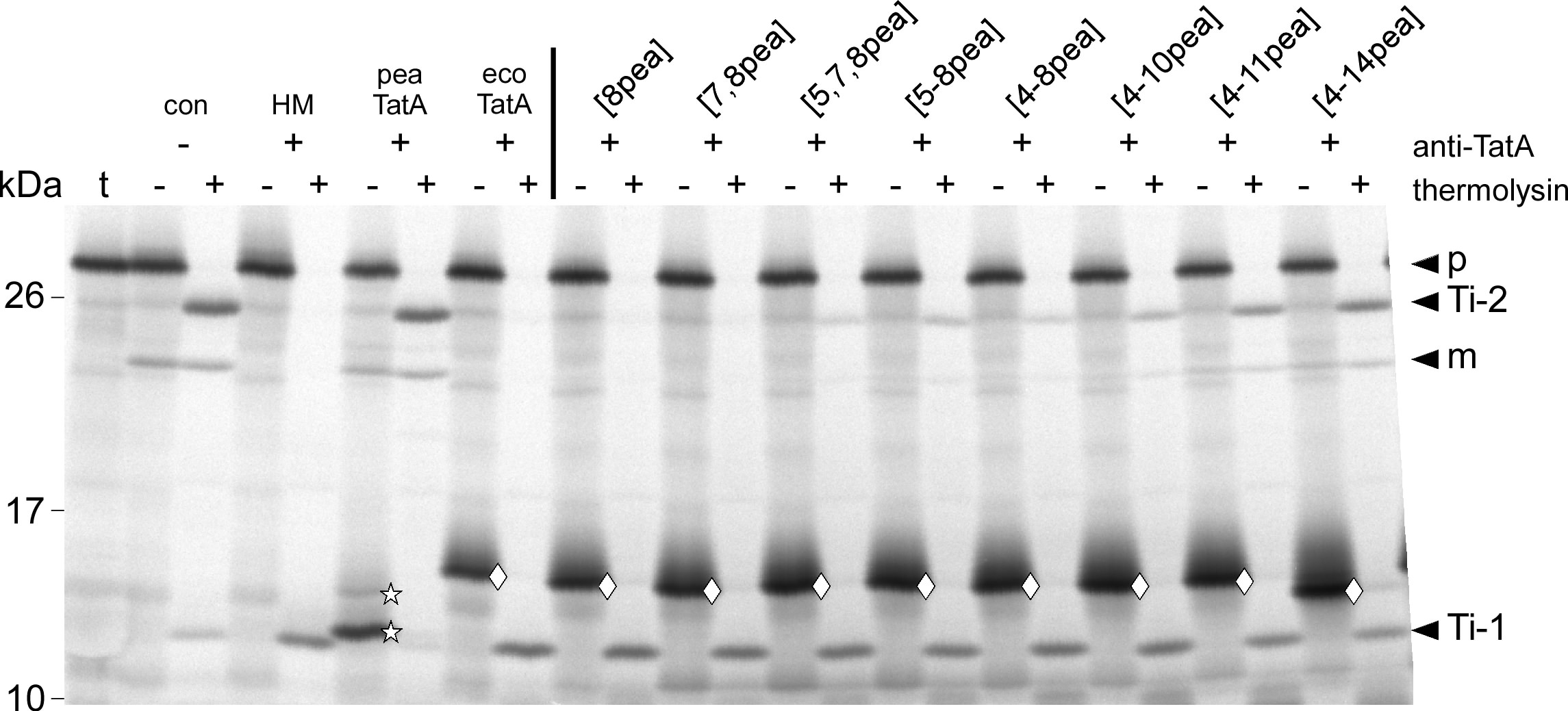
**

**Figure S4** *Active and inactive TatA chimeras show comparable binding to thylakoid membranes.*

*In thylakoido* complementation assays analysing the chimeric TatA proteins shown in Fig. 6A. The figure represents the complete gel shown in Fig. 6B. The TatA proteins were obtained from *in vitro* translation in the RTS system in the presence of [^35^S]-methionine, which allows their detection by autoradiography (stars: peaTatA, diamond: ecoTatA and chimeric TatA proteins). For further details see the legends to Figs. 1 - 3.


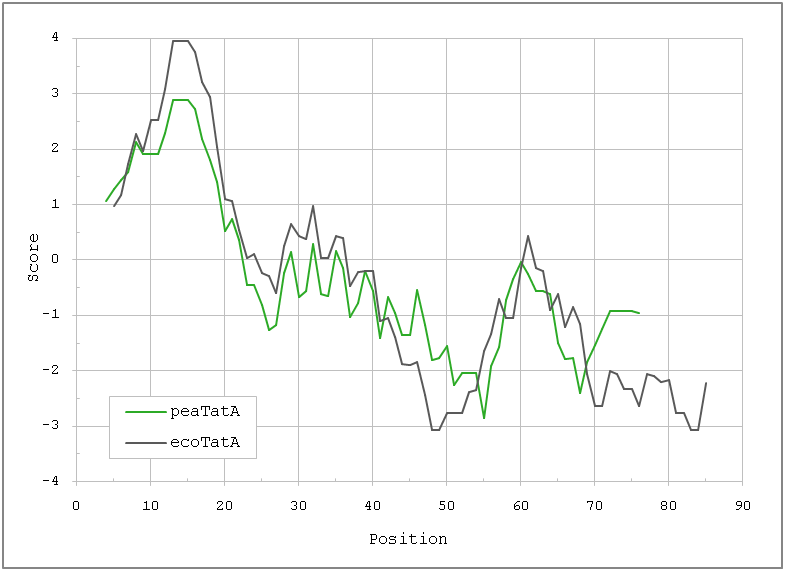


**Figure S5** *Hydropathy analysis of TatA proteins from pea and E. coli.*

Hydropathy analysis of peaTatA (*green line*) and ecoTatA (*black line*) according to Kyte & Doolittle^56^ using an average moving interval of 9 residues. The graph was adapted to the aligned amino acid sequences (Fig. 1) to take into account the deviating sizes of the two N-terminal regions.

Supplementary Reference:

56. Kyte, J. & Doolittle, R. F. A simple method for displaying the hydropathic character of a protein. J. Mol. Biol. 157, 105–132 (1982).

**Table S1** Primer sequences used in this study

| Primer | Forward (5´→ 3´) | Reverse (5´→ 3´) |
| --- | --- | --- |
| peaTatA | CTTTAAGAAGGAGATATACCATGGCCTTCTTCGGTCTAGGC | GATGAGAACCCCCCCCGGGTTATCATACATTATCCTTTGTG |
| ecoTatA | TGACCATGGATGGGTGGTATCAGTATT | TAGCCCGGGTTACACCTGCTCTTTATC |
|  |  |  |
| ecoTatA[N22pea] TMH+HR | AATCAGGAACAGGCTAAAACAGAAGAC | TTTCTTGGGACCGAAAACAAGAG |
| ecoTatA[N22pea] APH+CTD | GAGCCGAGGTAGCCAAGGCTAG | TAAAATTGTTTTATAATTGCGAATGTTAAAGG |
|  |  |  |
| ecoTatA[N19pea] | GCTCTTGTTTTCGGTACCAAGAAACTCGGC | GCCGAGTTTCTTGGTACCGAAAACAAGAGC |
| ecoTatA[N17pea] | GCTCTTCTTTTCGGTACCAAGAAACTCGGC | GCCGAGTTTCTTGGTACCGAAAAGAAGAGC |
| ecoTatA[N16pea] | GCGGGAGTCGCTGTTCTTCTTTTCGG | CCGAAAAGAAGAACAGCGACTCCCGC |
| ecoTatA[N15pea] | GTTATTGCGGGAGTCGTTGTTCTTCTTTTCGG | CCGAAAAGAAGAACAACGACTCCCGCAATAAC |
| ecoTatA[N14pea] | CTTGTTGTTATTGCGGGAATCGTTGTTCTTCTTTTCG | CGAAAAGAAGAACAACGATTCCCGCAATAACAACAAG |
| ecoTatA[N11pea] | GAGCTTGTTGTTATTGCGGTAATCGTTGTTCTTCTTTTCG | CGAAAAGAAGAACAACGATTACCGCAATAACAACAAGCTC |
| ecoTatA[N10pea] | GTGCCTGAGCTTGTTATTATTGCGGTAATCG | CGATTACCGCAATAATAACAAGCTCAGGCAC |
| ecoTatA[N8pea] | CTAGGCGTGCCTGAGCTTCTTATTATTGCGGTAATC | GATTACCGCAATAATAAGAAGCTCAGGCACGCCTAG |
| ecoTatA[N7pea] | GTCTAGGCGTGCCTCAGCTTCTTATTATTG | CAATAATAAGAAGCTGAGGCACGCCTAGAC |
|  |  |  |
| ecoTatA[8pea] | GTGGTATCAGTATTTGGGAGTTATTGATTATTGCCGTC | GACGGCAATAATCAATAACTCCCAAATACTGATACCAC |
| ecoTatA[7,8pea] | TGGGTGGTATCAGTATTCCGGAGTTATTGATTATTGC | GCAATAATCAATAACTCCGGAATACTGATACCACCCA |
| ecoTatA[5,7,8pea] | GTCGATGGGTGGTATCGGTATTCCGGAGTTATTG | CAATAACTCCGGAATACCGATACCACCCATCGAC |
| ecoTatA[5-8pea] | GGGTGGTATCGGTGTTCCGGAGTTATTG | CAATAACTCCGGAACACCGATACCACCC |
| ecoTatA[4-8pea] | CAGCTTGTCGATGGGTGGTCTCGGTGTTCCGGAGTTATTG | CAATAACTCCGGAACACCGAGACCACCCATCGACAAGCTG |
| ecoTatA[4-10pea] | CTCGGCGTTCCGGAGTTAGTTATTATTGCCGTCATCGTTG | CAACGATGACGGCAATAATAACTAACTCCGGAACGCCGAG |
| ecoTatA[4-11pea] | GTTCCGGAGTTAGTTGTTATTGCCGTCATC | GATGACGGCAATAACAACTAACTCCGGAAC |
| ecoTatA[4-14pea] | GAGTTAGTTGTTATTGCCGGAATCGTTGTACTGCTTTTTG | CAAAAAGCAGTACAACGATTCCGGCAATAACAACTAACTC |
|  |  |  |
| AE FW BamHI | GAGAGGATCCACAGAGGAACATGTATGGCCTTCTTCGGTCTAGG |  |
| AE Rev EcoRV |  | GAGAGATATCAAACAAGGATTACACCTGCTCTTTATCGTG |
